# Problems with the nested granularity of feature domains in bioinformatics: the eXtasy case

**DOI:** 10.1186/1471-2105-16-S4-S2

**Published:** 2015-02-23

**Authors:** Dusan Popovic, Alejandro Sifrim, Jesse Davis, Yves Moreau, Bart De Moor

**Affiliations:** 1 KU Leuven, Department of Electrical Engineering (ESAT), STADIUS Center for Dynamical Systems, Signal Processing and Data Analytics, Kasteelpark Arenberg 10 box 2446, B-3001, Leuven, Belgium; 2 iMinds Medical IT, Kasteelpark Arenberg 10 box 2446, B-3001, Leuven, Belgium; 3 Wellcome Trust Sanger Institute, Wellcome Trust Genome Campus, CB10 1SA, Hinxton Cambridge, UK; 4 KU Leuven, Department of Computer Science, Celestijnenlaan 200A, B-3001, Leuven, Belgium

**Keywords:** data granularity, validation bias, learning bias, hierarchical sampling, bootstrapping, eXtasy, Random forest, ensemble classifiers

## Abstract

**Background:**

Data from biomedical domains often have an inherit hierarchical structure. As this structure is usually implicit, its existence can be overlooked by practitioners interested in constructing and evaluating predictive models from such data. Ignoring these constructs leads to potentially problematic and the routinely unrecognized bias in the models and results. In this work, we discuss this bias in detail and propose a simple, sampling-based solution for it. Next, we explore its sources and extent on synthetic data. Finally, we demonstrate how the state-of-the-art variant prioritization framework, eXtasy, benefits from using the described approach in its Random forest-based core classification model.

**Results and conclusions:**

The conducted simulations clearly indicate that the heterogeneous granularity of feature domains poses significant problems for both the standard Random forest classifier and a modification that relies on stratified bootstrapping. Conversely, using the proposed sampling scheme when training the classifier mitigates the described bias. Furthermore, when applied to the eXtasy data under a realistic class distribution scenario, a Random forest learned using the proposed sampling scheme displays much better precision that its standard version, without degrading recall. Moreover, the largest performance gains are achieved in the most important part of the operating range: the top of prioritized gene list.

## Background

The data resulting from biomedical experiments often forms an implicit hierarchy in terms of granularity. A typical example is genomic data, where a single gene can harbor many mutations while it is at the same time a part of higher-order constructs (e.g., chromosome, patient). Likewise, a modeled target variable reflects this intrinsic property of the data. These structures pose no significant problem if the granularity of a target matches that of a data record. However, this is often not the case as the values of features from different levels of the hierarchy could be mixed together to form data records, while the outcome might remain implicitly defined over coarser domain. In these situations, a flexible enough learning algorithm can be trained to "recognize" higher order structure by using only its corresponding features. This deficiency of learning can affect both performance of the prediction and correctness of the validation procedure.

**Figure 1:**
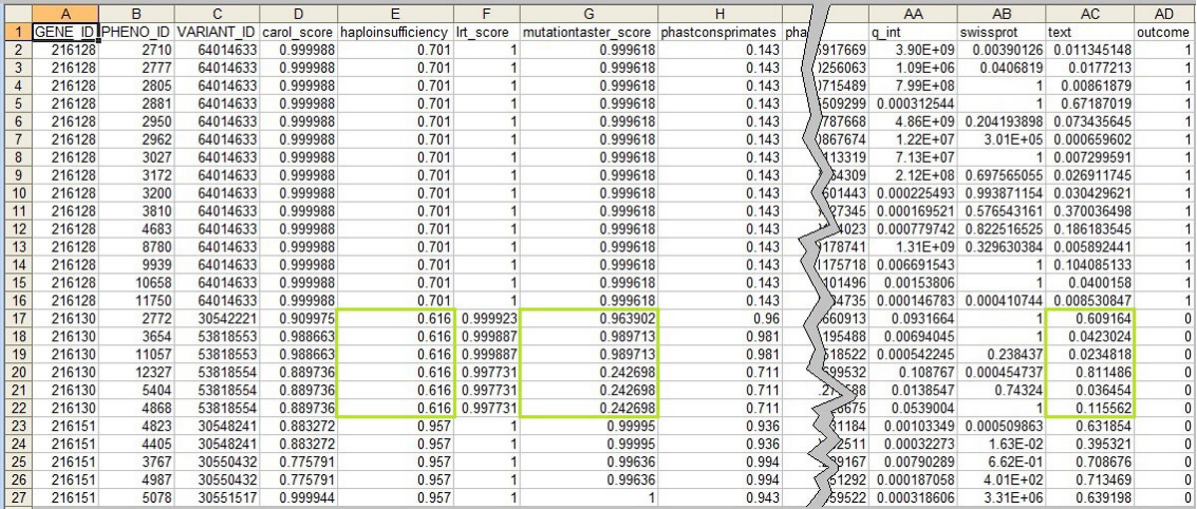
**A part of the eXtasy data**. Rectangles enclose values of haploinsufficiency (gene-level feature), mutation taster score (mutation-level feature) and a text mining based score from the Endeavor (gene+phenotype level feature). Note that all mutations within the gene associated with these data records are non disease-causing.

We illustrate the described issue on the case of the state-of-the-art variant prioritization algorithm called eXtasy [1]. This method is based on fusing genomic data and it incorporates predictors defined over three distinct levels of data granularity. On the gene level (the coarsest grain) it integrates the haploinsufficiency scores [2]. On the intermediate level, each mutation within a single gene is characterized by several additional mutation-level features including various deleteriousness prediction scores (Polyphen [3], SIFT [4], MutationTaster [5], LRT [6], CAROL [7], which are all extracted from the dbNSFP database [8]) and conservation scores for vertebrate, placental mammals, and primate groups [9,10]. Finally, a data record (the finest grain) is defined over mutation and a disease phenotype pair. It also contains additional features corresponding to diverse metrics obtained from the Endeavor variant prioritization tool [11]. The fact that Endeavor provides scores for gene/phenotype pairs makes the problem even more complex. Figure 1 displays a part of the eXtasy data where the described hierarchy of granularity is clearly visible.

One issue is how to evaluate predictive models learned from this kind of data. In this particular case all data records that correspond to the same mutation have an identical outcome: disease causing or not. If the data were randomly divided into training and validation sets on the instance level (i.e., without considering the hierarchical structure), an algorithm could achieve high accuracy by simply learning the SIFT score value for each mutation. In other words, the SIFT value acts as an unique identifier. This clearly distorts a validation scheme's reliability and leads to optimistically biased performance estimates. Fortunately, the aforementioned problem is relatively easy to alleviate: the partition scheme must ensure that all data instances sharing the same mutation ID either only appear in the training set or only appear in the validation set.

However, the gene level information is also present in the data records while most (but not always all!) mutations within a single gene share the same outcome. If the percentage of mutations that have different labels within a gene is smaller than the theoretically achievable classification accuracy, the validation could be again optimistically biased due to the previously described issue. If this is the case, the previous reasoning implies that the partition should be based on the gene as opposed to the mutation ID. This had been done during the initial eXtasy evaluation. However, sometimes the higher-order structures either are not obvious or are not known beforehand. In these cases, only a careful examination of the feature values can reveal potential problems.

The problem of inflated performance estimates in the presence of hierarchical feature granularity has been implicitly recognized before. One example is in the multicentric study that addresses the prediction of stroke patients restitution [12]. In this work, the authors noticed that when the data from several clinics are mixed together and then divided for the purpose of validation, the resulting performance is an overestimate compared to a genuinely external data set. To circumvent this, they advocate a "leave-one-center-out" validation approach. Note that in this case there was not a single center-level variable in the data, yet other variables, such as demographics, can serve as proxy for center-level *information*.

Similarly, a "leave-one-drug-subclass-out" approach was used to assess the prediction of the functional class of an unrepresented drug type [13]. There each model has been trained on all but one subclass of antidepressant drugs and then used to classify members of the subclass that was omitted during training. Furthermore, a "leave-one-cow-out" strategy was proposed in the context of predicting the somatic cell count in whole milk from near infrared spectroscopy measurements [14]. Surprisingly, this validation problem is not always fully recognized.

The second issue associated with hierarchical granularity is much more latent and therefore often neglected in the data modeling. That is, even if the validation scheme is appropriately defined with the respect to the highest level of data granularity on which the decision values are (mostly) uniform, the discussed bias still exists. In such a situation, performance estimates will be correct but the performance itself might decline. This happens if the rows (i.e., examples) are inter-independent, even though the data consists of a single table where each example is described as a fixed-length feature vector. The interdependencies exhibit themselves on different levels of granularity, where all inter-dependent examples have an identical value for a specific feature as well as the same value for the target variable. Thus the feature value appears correlated with the target variable whereas in reality the feature value is correlated with the hierarchical structure of the data. Failing to consider the interdependencies during learning could cause the algorithm to produce a model that simply identifies a pattern that is correlated with the hierarchical structure of the data as opposed to a pattern that is correlated with the target variable. In other words, the algorithm can be still overfitting on coarse grained features of the training data partition, failing to actually generalize from it.

In the context of the eXtasy data, the described bias materializes as learning, to a certain degree, to recognize genes which constitute the training set, instead of extracting general characteristics of disease causing mutations, leading to a reduced performance on the test set. This happens because many data instances share the same values of higher order features. For example, instead of learning that "mutation A that occurs in gene B is probably disease causing due to the high haploinsufficiency score of gene B (which implies correlation of haploinsufficiency with outcome)," the algorithm may infer that "mutation A is probably disease causing because the haploinsufficiency score of gene B is exactly 0.998 (which is a value that uniquely identifies the given gene)." The second rule does not provide any insight into a new example with haploinsufficiency of 0.999.

This situation is radically different from having one or more categorical variables in the data set, although sometimes it might be hard to distinguish between these two. While categorical features can also have identical values for several data instances and can be correlated with the outcome, their values are *not ordered*. Thus, here it may be desirable that the algorithm learns the correlation between particular categories and the outcome, instead of extracting some trend that involves the ranges of the variable values. In addition, when the trained model is applied on unseen data the number of possible categories typically stays the same. This is not the case with coarse-grained features, as previously unobserved values of these variables are often present in new data (ex. a SIFT score of previously undiscovered mutation that did not participate in training).

In the remaining text, we describe a simple, sampling-based method for dealing with the described bias. First, we further illustrate the problem using a conveniently generated synthetic data set and analyze the robustness of the solution to different factors, including the grain size and the level of label noise. Second, we demonstrate how the performance of the core eXtasy model can be improved through the use of the hierarchical sampling.

## Methods

### Hierarchical sampling

The core model of eXtasy is based on the Random forest classifier [15]. Random forests are an ensemble method that constructs a set of unpruned decision trees using different bootstrap samples [16] of the data. Furthermore, randomness is injected during model construction by only considering a randomly sampled subset of the candidate variables at each split point, as opposed to scanning all candidate variables as is done in traditional decision tree learning. The final label of an unseen example is an unweighted vote of the predictions by each model in the ensemble (as done in bagging [17]). Hence, Random forests may be particularly susceptible to the described problem due to the fact that they both sample examples and attributes. That is, failing to account for the correlations during this sampling could produce a biased sample.

We propose a straightforward modification to the Random forest framework, named hierarchical sampling. Instead of extracting a bootstrap from the complete training set to build a single tree on, we first stratify the training examples according to the distinct values of the feature over which the coarsest granularity level is defined. In the case of the eXtasy data, that would be the gene identifier. Note that a feature could implicitly define the grain while not being a part of a data set at all (a "latent factor" of the outcome grouping), suggesting that domain knowledge is needed to perform this procedure. After stratification, we randomly select just one data instance from each partition to form the in-bag sample for learning one tree. This helps prevent a single tree from learning to recognize a particular value of the higher-order feature, as only one example having the value will be present in each sample. At the same time, each partition will be well represented in the ensemble as a whole, provided that a sufficient number of trees are learned. Algorithm 1 formally describes this procedure.

**Algorithm 1 **Random Forest with hierarchical sampling

Inputs:

*D *= {*P*_1 _= {*e*_1,1_,...,*e*_1,*n*_},...,*P_p _*= {*e*_*p*,1_,...,*e_p,m_*}} is the set of training examples, divided into *p *partitions, where each partition corresponds to examples sharing the same value of the feature that is defined over the coarsest domain

*t *is the number of trees to learn

1: Let *T *= ∅

2: **for ***i *= 0 to *t ***do**

3:   Let *BS *= ∅

4:   **for ***j *= 0 to *p ***do**

5:      Let *k *~ Uniform[0, |*Pj*|]

6:      *BS *= *BS *∪ {*e_j,k_*}

7:   **end for**

8:   *t_i _*= LearnTree(*BS*)

9:   *T *= *T *∪ {*t_i_*}

10: **end for**

11: **return ***T*

**Algorithm 2 **Random Forest with stratified bootstrapping

Inputs:

*D *= {*P*_1 _= {*e*_1,1_,...,*e*_1,*n*_},...,*P_p _*= {*e*_*p*,1_,...,*e_p,m_*}} is the set of training examples, divided into *p *partitions, where each partition corresponds to examples sharing the same value of the feature that is defined over the coarsest domain

*t *is the number of trees to learn

1: Let *T *= ∅

2: **for ***i *= 0 to *t ***do**

3:   Let *BS *= ∅

4:   **for ***j *= 0 to *p ***do**

5:      **for ***k *= 0 to |*P_j_*| **do**

6:         Let *l *~ Uniform[0, |*P_j_*|]

7:         *BS *= *BS *∪ {*e_j,l_*}

8:      **end for**

9:   **end for**

10:   *t*_*i *_= LearnTree(*BS*)

11:   *T *= *T *∪ {*t_i_*}

12: **end for**

13: **return ***T*

It is important to distinguish the described procedure from stratification in the classical sense as outlined in Algorithm 2. While the later is often used with the Random forest classifier and seems very similar to the hierarchical sampling, its purpose is diametrically different. The stratification is typically used for making sure that each of the (latent) classes (in our case examples having the same value of the coarsest feature) is sufficiently and accurately represented. This is usually achieved by bootstrapping each stratum separately, in contrast to taking just one example from it. Hence, while this procedure is appropriate in many situations, such as when dealing with categorical variables, in this given context classical stratification can lead to overfitting by guarantying the repeated presence of each gene within the in-bag data partition of each tree in the ensemble. Conversely, taking one example per stratum assures that no gene-level information will be over-represented in a single tree. Repeating the sampling before learning each new tree helps to protect the model from under representing the data as *a whole *in the *ensemble*. To further underline this distinction, we include a Random Forest model trained with stratification in the following synthetic data analyses.

### Experiments with synthetic data

**Figure 2:**
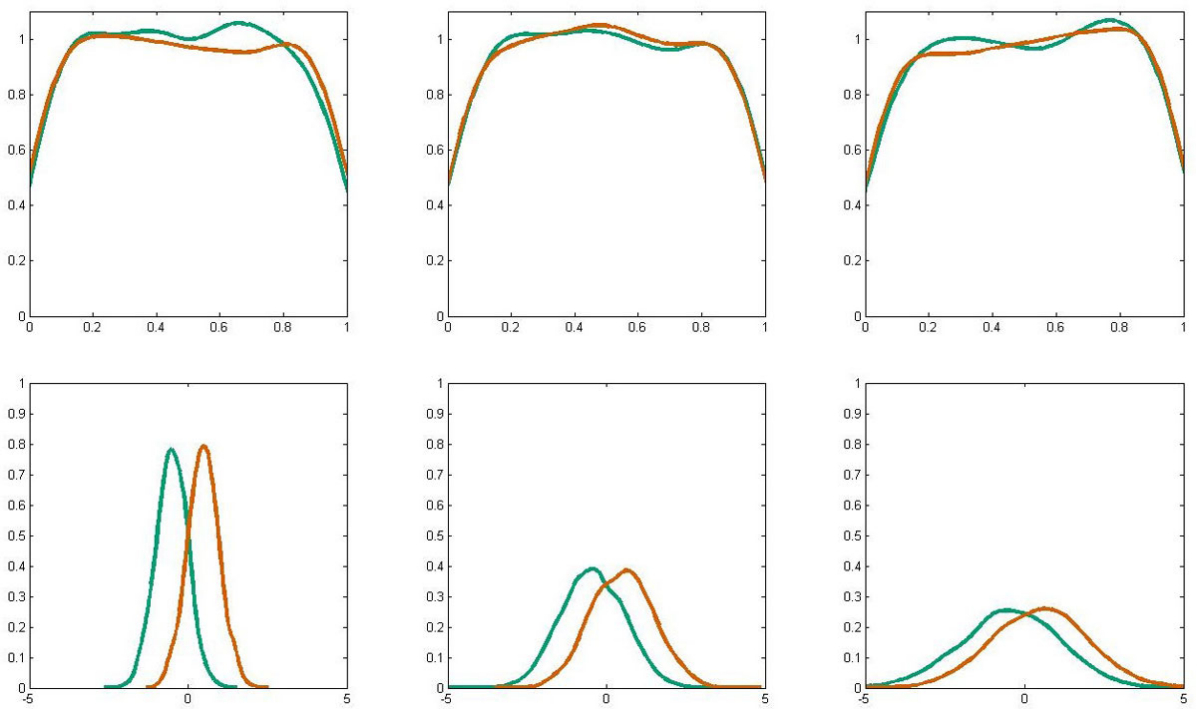
**Features of the synthetic data**. Kernel density estimates of the synthetic data distributions. Panels in the first row display distributions of three *non-informative *features for two classes separately. Note that decreased density in the vicinity of sampling interval borders is in fact an artifact of the kernel density estimation procedure and not of the uniform sampling itself, thus it affects only this visualization. The bottom three panels depict *informative *features.

To characterize the discussed bias in greater detail and to analyze its sources and extent, we constructed synthetic data where the phenomena is isolated from other sources of variation and is relatively easy to control. This data set is described by seven variables and it has a binary class label. We generated four thousand examples such that the class distribution is balanced (i.e., that data have two thousands examples for each class). The first three features are *non-informative* and are simply sampled uniformly from the interval (0,1). The next three features are *informative *and each one is represented by a conditional Gaussian distribution. The mean depends on the class and the variance, and hence the overlap, is different for each feature (see Figure 2). The first six features remain unchanged in all experiments. The seventh feature has been created to represent the hierarchical structure in the data. It will be manipulated in the two experiments.

The first one has been designed to reveal the relation between the size of the coarsest variable partitions (grain) and magnitude of the described bias. In particular, the additional *non-informative *feature has been generated by sampling its value uniformly and appending it to the rest of the data, as done previously. However, in this case the number of distinctive values that this new feature can take has been varied from two to two thousands per class, which led to a change in granularity of the feature domain. In total, ten possible sizes of partition have been considered (|*P*| ∈ {1, 2, 5, 10, 20, 50, 100, 200, 500, 1000}).

Given each of these, the appropriate feature has been created, after which the whole data set has been divided into equally sized training and testing partitions along its distinct values. All three classification models are then learned using the training data and evaluated on the test data. The total number of trees in each model ensemble has been set to 1000 and the size of the random subset of variables from which each decision tree split is chosen (parameter *M*) to its recommended value for classification, that is, to the square root of the total number of features [15]. This whole procedure has been repeated one hundred times for every bin size. In each repetition, the auxiliary feature is regenerated and a new, random training-test split is made. In addition, in order to facilitate complementary insight into the learning process, the out-of-bag feature importance measures [15] obtained from the classical Random forest model have been harvested along the way.

The second experiment will assess the effect of label noise on the extent of the bias. The label noise was introduced by swapping certain percentages of the values from each partition of the auxiliary feature belonging to the one class with values of its counterpart from the other class. This percentage is varied from 5 to 50% in steps of 5, resulting in ten possible noise levels in total. The partition size was increasing again, but in this case starting from 20 to allow for the meaningful injection of noise. Finally, training and evaluating the models have been performed in the same way and with the same parameter setting as in the previous experiment.

### Experiments with eXtasy data

To ensure a fair comparison, we test the method on the original eXtasy benchmark data. Briefly, this data set consists of two classes of mutations: disease causing variants and rare mutations present in healthy individuals. There are 24,454 disease causing variants in the data set, which were obtained from the Human Gene Mutation Database (HGMD [18]). The controls come from 68 in-house sequenced exomes of healthy individuals and the 1000 Genomes Project [19]. For each of the 1142 Human Phenotype Ontology (HPO) terms associated with the disease-causing variants, 500 mutations were randomly sampled from the pool of controls and assigned to a given phenotype. Endeavor scores [11] have been appended to each variant-phenotype combination, together with haploinsufficiency [2], conservation [9,10] and deleteriousness prediction scores [3-6].

To compare hierarchical sampling to the standard bootstrapping, we use the same evaluation scheme as in the original study [1]. That is, we randomly divide the complete benchmark data set on the gene-level such that two-thirds of the genes constitute the training set and one-third are in the test set. We consider two test scenarios. In the first one, we compare two sampling schemes on the unaltered test set, effectively repeating the eXtasy benchmark. In the second one, we randomly undersample the positives from the test set in order to mimic the class distributions we would expect to see in the wild, where only one out of 9000 non-synonymous mutation in a genome is potentially disease-causing [20]. In both scenarios, and for both sampling schemes, we use the same setting for the Random forest parameters as in the synthetic data experiments. We repeat the aforementioned procedure 100 times to stabilize the values of the performance metrics.

Finally, we evaluate the significance of the observed differences between the two methods by using the Wilcoxon signed-ranks test together with the Bonferroni multiple testing correction. When comparing two classifiers, the Wilcoxon signed-ranks test is preferred to the standard t-test [21], because the later exhibits a high probability of type I error when used in this context [22]. Similarly, the Bonferroni correction is very conservative [23], so we use it to stay on a safe side when making an inference about differences in performance.

## Results and discussion

**Figure 3:**
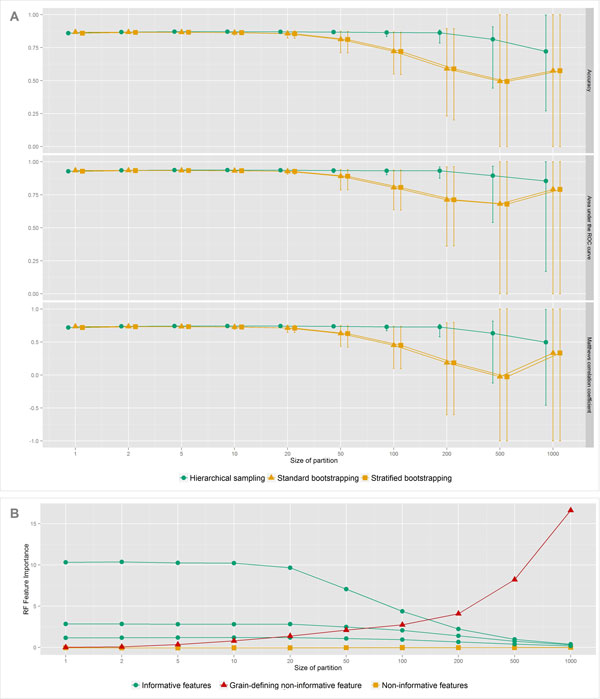
**Results of the first experiment on the synthetic data**. Evolution of testing accuracy, area under the ROC curve and Mathews correlation coefficient of Random forest classifiers trained with three sampling schemes and under the increasing size of partitions of the grain-defining feature (Panel A). Vertical bars indicate empirical 95% confidence intervals. Panel B displays corresponding change in Random forest feature importance metrics for all features of the synthetic data set under the standard bootstrapping. This metric captures an increase in out-of-bag classification error when the values of given feature are shuffled.

**Figure 4:**
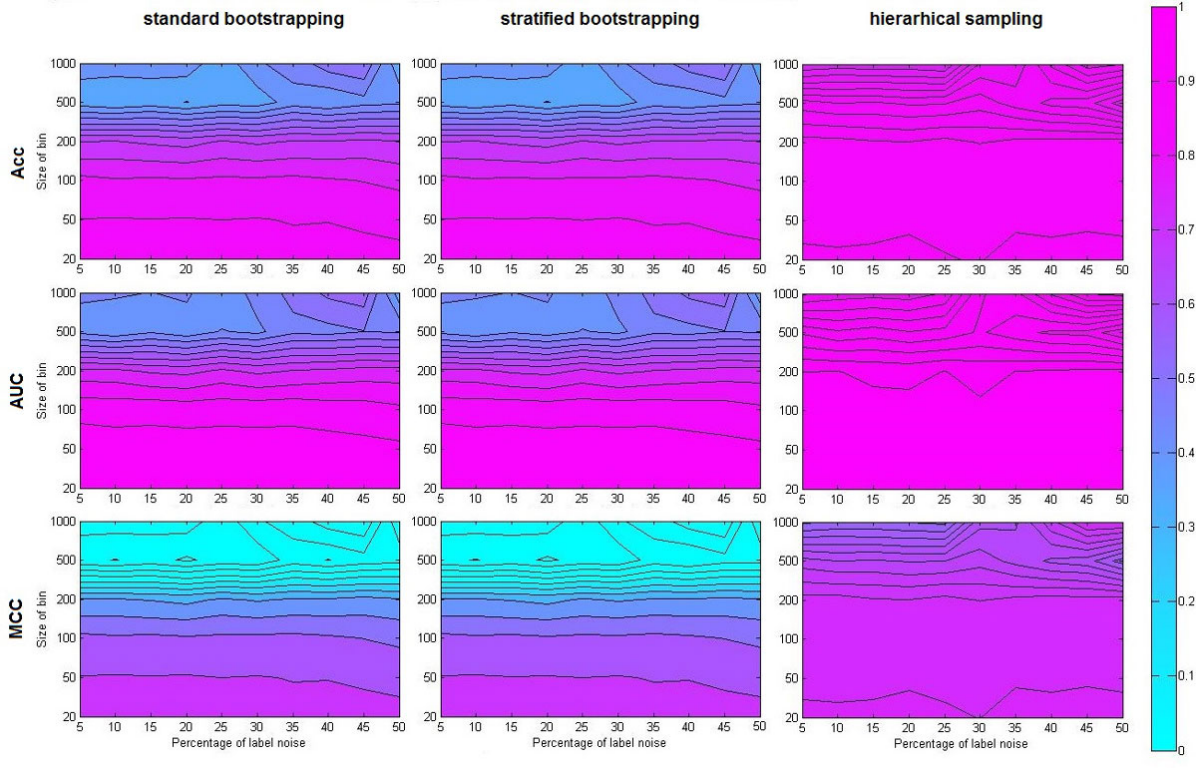
**Results of the second experiment on the synthetic data**. Heat-maps of mean testing accuracy (Acc, the first row), area under the ROC curve (AUC, the second row) and Mathews correlation coefficient (MCC, the third row) of Random forest classifiers trained with either standard bootstrapping (the first column), stratified bootstrapping (the second column) or hierarchical sampling (the third column). Panels capture relation between given performance metrics and bin size/label noise level combinations. Note that values of Mathews correlation coefficient can be as low as -1, which is the reason why upper parts of corresponding plots have uniform coloring (i.e. all values in this region are smaller than zero).

The results of the synthetic data benchmarks are provided in Figures 3 and 4. Due to space limitations and the class-balanced design of the synthetic data, we only present aggregate measures for classification performance. From Figure 3, it is immediately apparent that increasing the partition size directly leads to a decrease in the performance of the classical Random Forest formulation, starting from bin sizes as small as twenty. This trend is evident even in this setup, where we do not make smooth transition between the number of instances in a partition.

The evolution of the associated feature importance metrics suggests that the reason for the decreased performance is indeed progressive overfitting on the additional non-informative feature. That is, in parallel with bin enlargement, this feature becomes increasingly important for classification, while at the same time the importance of the genuinely informative features decline. Furthermore, training the same classifier with stratification does not seem to help, as its performance tightly follows that of the regular version.

Conversely, the Random Forest trained with hierarchical sampling stays relatively insensitive to the change in bin size, with the only exception being the last step. However, this is a degenerate case in which the method has only one example per class available for training each tree, which is clearly insufficient for good generalization. Also, this situation is not likely to be encountered in practice. The described effect persists even in the presence of label noise, as it is apparent in Figure 4. Yet in the case of the classic Random forest or the Random forest trained with stratified bootstrapping, the bias obviously declines as the amount of added noise is increased. This is expected, as adding label noise translates to an implicit reduction of the partition size.

**Figure 5:**
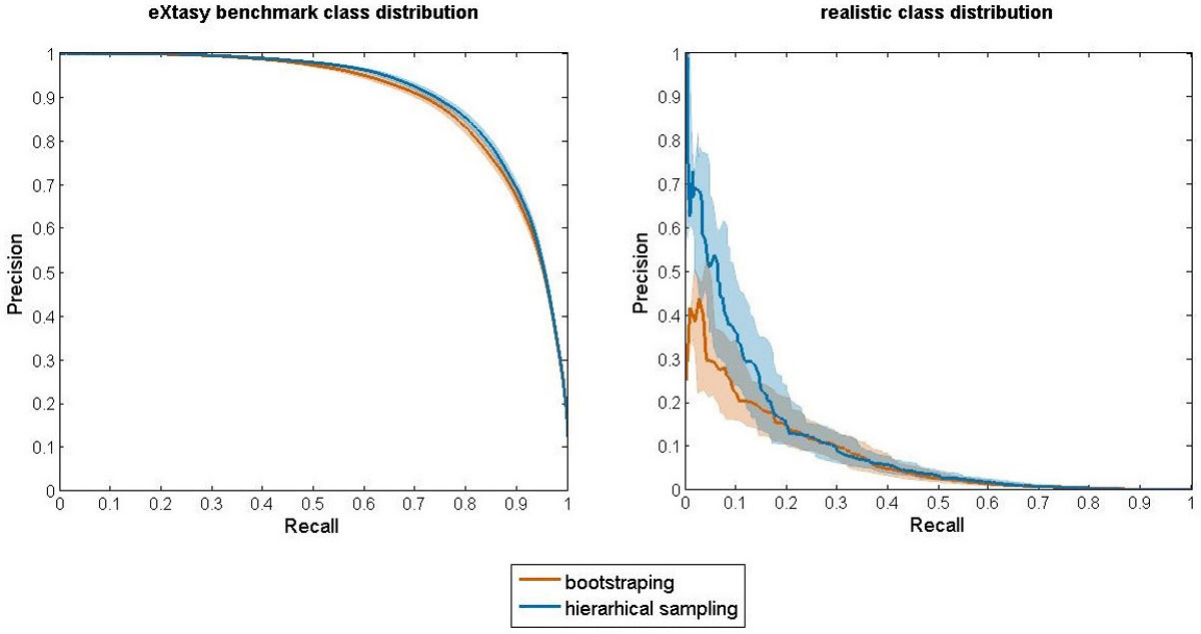
**PR curves from eXtasy-based experiment**. Precision-recall (PR) curves obtained by application of the eXtasy on the data with class balance as in original eXtasy benchmark (left panel) and on the data with realistic class distribution (right panel). Each panel display two solid curves - the one corresponding to the standard Random forest classifier training with bootstrapping and the one corresponding to hierarchical sampling based training. The shaded areas represent empirical 95% confidence intervals obtained as a by-product of threshold averaging.

The results of the eXtasy-based benchmark are provided in Table 1 and Figure 5. Figure 5 shows the PR (Precision-Recall) curves only, as there the difference in performance between the two methods is graphically evident. These curves are constructed using threshold averaging [24] over the 100 runs. Table 1 reports the corresponding area under the curve values. Following the recommendations for reporting on classification benchmarks [25] and to provide insight into the different aspects of the classification performance, Table 1 also provides average values for Precision (Positive predictive value), Recall (Sensitivity), Negative predictive value, Specificity and the Matthews correlation coefficient for both scenarios and indicates if the differences are statistical significant. In addition, it provides values for the area under the ROC (Receiver Operating Characteristics) curves obtained by threshold averaging.

**Table 1:** Results of the eXtasy-based benchmark.

	eXtasy benchmark class distribution		realistic class distribution	
**Metric**	**bootstraping**	**hierarchical sampling**	**bootstraping**	**hierarchical sampling**

Sensitivity	0.863751*	0.783985	0.888667*	0.816667
Specificity	0.951801	0.980001*	0.951809	0.980030*
Precision	0.711571	0.842510*	0.002423	0.005313*
NPV	0.980934*	0.970764	0.999985*	0.999976
MCC	0.751416	0.788129*	0.044896	0.064834*
AUC-ROC	0.972466	**0.973497**	0.979336	**0.981345**
AUC-PR	0.888809	**0.895255**	0.095083	**0.128191**

Results of the benchmark in terms of average Sensitivity, Specificity, Precision, Negative predictive value (NPV) and Matthews correlation coefficient (MCC). Bold typing indicates which method of generating in-bag sample is better given a performance measure and a classification scenario, while asterisk (*) points on statistically significant differences (p = 0.05 level). The values of the area under the ROC and PR curve are obtained from the average curves constructed by threshold averaging; hence no statistical testing has been applied on these.

The difference between bootstrapping and hierarchical sampling in terms of the achieved area under the ROC and PR curve might seem marginal when only the result on the eXtasy benchmark data is considered. However, the realistic class distribution scenario highlights the benefit of hierarchical sampling as there are large improvements in PR space, especially in the low-recall, high-precision region of curve which is of most interest for prioritization algorithms. Due to the associated financial costs of confirmatory experiments, analysis is usually only conducted on highly ranked candidate variants (i.e., those at the top of the ordered list) and not on all mutations that are classified as positive. Thus the precision of the prioritization method for the highest ranked genes is of critical importance, especially as most state-of-the art deleteriousness prediction methods suffer from high false positive rates when predicting the impact of rare disease-causing mutations [26].

The discussed performance differences are difficult to detect when using ROC analysis alone, as ROC curves are invariant to changes in the class distribution. This property of ROC curves is also the reason for which they should, in principle, be identical for both *scenarios*, given a classifier. Naturally, they are not, due to variation in performance estimates. This variance is large enough to also cast doubt on the systematic cause of any observed difference in ROC curves between *classifiers *in the same scenario. However, dominance of one curve over another in PR space implies dominance in ROC space as well [27], while the *size *of the effect can differ. Hence, the extent of the difference between two classifiers in PR space for the realistic class distribution scenario constitutes indirect proof of the existence of the systematic effect in ROC space.

As the result in the table show, the hierarchical sampling RF performs worse than the standard RF in terms of sensitivity in both test scenarios. Conversely, this is offset by the higher precision it achieves in both settings. Being complementary to the measures already mentioned, specificity and the negative predictive value also reflect the aforementioned trade-off, albeit to a smaller degree (as more abundant negatives play major role in these). However, as is apparent from the PR curves, setting the decision threshold of the RF trained using the hierarchical sampling so that it achieves the same sensitivity of the standard RF still results in the higher precision of the first RF. These results are in line with previous discussions on differences in performance at the top of prioritized lists. Additionally, even with the default RF threshold (0.5), the improved version of eXtasy classifies approximately 188 out of 9000 variants as disease causing, with the probability of capturing the real one equal to 0.81 (i.e. sensitivity). In contrast, the standard eXtasy calls 417 out of 9000 variants, with the probability of hit being 0.88.

We acknowledge that the presented results may vary with the information content of the grain-defining feature and the rest of predictors, correlations among them and the other factors. However, due to difficulties with quantifying these aspects and the exponential number of potential relations among them, we omit their detailed treatment from this work. We also expect that further improvements in performance could be obtained by fine tuning the parameter *M *of the Random forest algorithm. That is, the application of hierarchical sampling increases the diversity of an ensemble compared to the standard bootstrapping, possibly allowing for larger values of *M *to be used [28]. Bigger values of *M *might translate to an improved accuracy for the base classifiers, resulting in an overall boost in performance.

## Conclusions

We described the bias that arises when learning from data characterized by nested granularity of the feature domains and proposed a simple solution for it, named hierarchical sampling. Using synthetic data, we demonstrated that the approach efficiently mitigates the discussed bias, regardless of the grain size and the amount of the label noise that is present in the data. The simulation study also indicated that the gain in performance can vary substantially for different classes of problems. That is, if the number of distinctive values of the coarsest grain concept is much smaller than total number of data records, overfitting on these concepts is more likely to occur. Furthermore, in the case of the eXtasy variant prioritization algorithm, the hierarchical sampling led to a notable improvement in the model performance in terms of the precision, especially in the most important operating regions for this particular application. Also, as it uses less data (per single tree) than standard Random forests bootstrapping, it typically results in a more parsimonious model in terms of the average tree depth.

However, the improvement in performance that might be obtained by using hierarchical sampling is only visible under proper evaluation. In particular, if a hierarchy of feature domains (even implicitly) exists in the data and it is neglected during the evaluation, standard Random forest will falsely perform better on the test data due to the evaluation problems described in the "Background" section of this manuscript. Conversely, a Random forest trained with the hierarchical sampling would not be able to memorize values of the grain-defining feature, and thus to recognize them in the test set. Hence the situations where the proposed approach can be useful are the same ones where the data partitioning for validation purpose must be performed along values of an implicit identifier having the lowest number of distinctive values.

Finally, even the application of hierarchical sampling is not in principle restricted to Random forests, the method can be efficiently used only in conjunction with an ensemble classifier that relies on some sort of bagging scheme. If sampling from the highest granularity level would be applied in the one-shot fashion (e.g., for training an SVM), much data would be ignored during training which could negatively affect performance. That is, even in cases where the sample itself is reasonably large, general information embedded in the fine-grained features might not be sufficiently represented. In contrast, bagging allows for all training data to be fully exploited, while the proposed sampling ensures that no base classifier can learn to recognize higher order structures in data. In the near future, we plan to integrate the Random forest model learned using the hierarchical sampling into the next version of the eXtasy framework.

## Competing interests

The authors declare that they have no competing interests.

## Authors' contributions

D.P. and A.S. conceptually defined the problem. D.P. proposed a solution, developed the benchmarks and performed the analyzes. D.P. and J.D. wrote the initial draft of the manuscript. A.S. generated the data sets. J.D. advised on general machine learning concerns. B.D.M. supervised the project. Y.M. co-supervised the project. All authors revised and proofread the manuscript.

## Supplementary Material

Additional file 1 **A Matlab function for training Random Forest with hierarchical sampling**. This Matlab function accepts training data, training outcomes, desired number of trees in the ensemble and the ordinal number of the feature over which the hierarchical sampling has to be performed, and returns a trained Random forest classier. Click here for file

## Declarations

The publication of this article is funded by the STADIUS research group of KU Leuven.

This article has been published as part of *BMC Bioinformatics *Volume 16 Supplement 4, 2015: Selected articles from the 9th IAPR conference on Pattern Recognition in Bioinformatics. The full contents of the supplement are available online at http://www.biomedcentral.com/bmcbioinformatics/supplements/16/S4.
